# Seasonal variations in food resource partitioning among four sympatric gudgeon species in the upper Yangtze River

**DOI:** 10.1002/ece3.5293

**Published:** 2019-05-28

**Authors:** Fei Liu, Jianwei Wang, Huanzhang Liu

**Affiliations:** ^1^ The Key Laboratory of Aquatic Biodiversity and Resource Conservation, Institute of Hydrobiology Chinese Academy of Science Wuhan Hubei Province China

**Keywords:** feeding habit, Gobioninae, resource partitioning, the upper Yangtze River

## Abstract

Knowledge of food resource partitioning among sympatric fish species is crucial for understanding the potential mechanisms of species coexistence. Gudgeons (Teleostei: Cyprinidae: Gobioninae) often dominate fish assemblages in the upper Yangtze River. However, little research has been conducted on their trophic interactions. In this paper, seasonal diet and feeding strategy variations of four sympatric gudgeon species, *Coreius guichenoti*, *Coreius heterodon*, *Rhinogobio ventralis,* and *Rhinogobio cylindricus*, were investigated by analysis of intestinal tract contents, aiming to explore whether food resource partitioning occurred among them. Fish specimens were collected during spring (April–May) and autumn (August–October) in 2010 in Hejiang, a free‐flowing stretch of the upper Yangtze River. *Coreius guichenoti*, *C. heterodon,* and *R. cylindricus* showed omnivorous feeding habits, while *R. ventralis* exhibited an obligate carnivore feeding habit. Diet overlap among the four studied species was high, especially in spring. However, changes in feeding strategies were observed in autumn. Specifically, *C. guichenoti* and *R. cylindricus* expanded their dietary niche breadth and consumed detritus, Sinopotamidae or Hydropsychidae as important complementary food resources. In contrast, *C. heterodon* and *R. ventralis* reduced their dietary niche breadth and became more specialized on mussels (*Limnoperna lacustris*). These results confirmed that sympatric fish species can coexist with high diet overlap, and food resource partitioning among these species may also fluctuate with the seasons.

## 1 INTRODUCTION

Knowledge of resource partitioning is essential to understand the potential mechanisms facilitating the coexistence of species with similar niches (Gabler & Amundsen, 2010; Juncos, Milano, Macchi, & Vigliano, 2015; Sánchez‐Hernández, Gabler, & Amundsen, 2016), and the understanding of these mechanisms is critical for developing effective conservation and management plans (Kallgren, Pedersen, & Nilssen, 2015). According to the competitive exclusion principle, species with similar ecological niches cannot coexist within the same ecological communities, because interspecific competition will lead to an exclusion of the competitively weaker species or a partitioning in resource utilization (Gause, 1934). Therefore, species that coexist harmoniously in the same communities are expected to segregate with respect to the use of food, space, and/or time (Schoener, 1974; Gabler & Amundsen, 1999; Pianka, 2000).

In aquatic ecosystems, food resource is considered as the most important driver for resource partitioning (Ross, 1986). Many studies have revealed that food resource partitioning among sympatric fish species can occur in different levels, and species can segregate in diet composition (Coelho, Martins, Collares‐pereira, Pires, & Cowx, 1997; Gray, Boltz, Kellogg, & Stauffer, 1997), prey size (Sánchez‐Hernández & Cobo, 2011; Sánchez‐Hernández, Vieira‐Lanero, Servia, & Cobo, 2011), diel activity patterns (Sánchez‐Hernández et al., 2011), and foraging water column (Hesthagen, Saksgård, Hegge, Dervo, & Skurdal, 2004; Olson, Jensen, & Hrabik, 2016; Sánchez‐Hernández et al., 2016) to reduce interspecific competition. For example, in studying the feeding relationships between two Iberian cyprinids in the Sorraia river system, Coelho et al. (1997) found that Iberian roach *Rutilus alburnoides* fed mainly on dipteran larva, whereas chub *Leuciscus pyrenaicus* consumed predominantly Ephemeroptera nymphs and imagines. Similarly, Sánchez‐Hernández et al. (2011) observed that the four sympatric fish species in the River Ladra can be classified into two trophic guilds: species fed mainly on detritus and plant material, and species fed mainly on aquatic macroinvertebrates.

Noteworthy, food resource partitioning patterns among sympatric fish species may change obviously across seasons, according to the seasonal variations in food availability, including food diversity and food abundance (Prejs & Prejs, 1987; Sánchez‐Hernández et al., 2016; Sánchez‐Hernández, Gabler, & Amundsen, 2017; Gray et al., 1997). Some researchers found that co‐occurring species may specialize following their species‐specific resource preferences when food resource become limited and the overlap will decrease (Gabler & Amundsen, 1999; Robinson & Wilson, 1994; Schoener, 1971; Gray et al., 1997). For example, Gray et al. (1997) observed greater trophic partitioning among sympatric fish species in April, when food resource was scarce, than in July, when prey was abundant. Deus and Petrere‐Junior (2003) noted that fish species were more generalized in summer when food availability was higher and more specialized in winter when food resource was scarce. In contrast, other researchers insisted that species should be forced to converge and to exploit the same resources when the food density was low (Liem, 1984; Magalhães, 1993; Pyke, Pulliam, & Charnov, 1977; Wiens, 1993). Under this circumstance, the population trophic niche breadth will expand and the overlap will increase (Liem, 1984; Magalhães, 1993; Pyke et al., 1977; Wiens, 1993). Sánchez‐Hernández et al. (2017) demonstrated that these seemingly contradictory standpoints can be solved when food diversity is taken into consideration. That is, alternatives to niche differentiation can be used to explain the coexisting of sympatric species (Amarasekare, 2003; Gabler & Amundsen, 2010; Genner, Turner, & Hawkins, 1999; Sánchez‐Hernández et al., 2017). However, more studies are recommended to examine what component of food availability (prey diversity and prey abundance) affects food resource partitioning among sympatric fish species (Sánchez‐Hernández et al., 2017).

**Figure 1:**
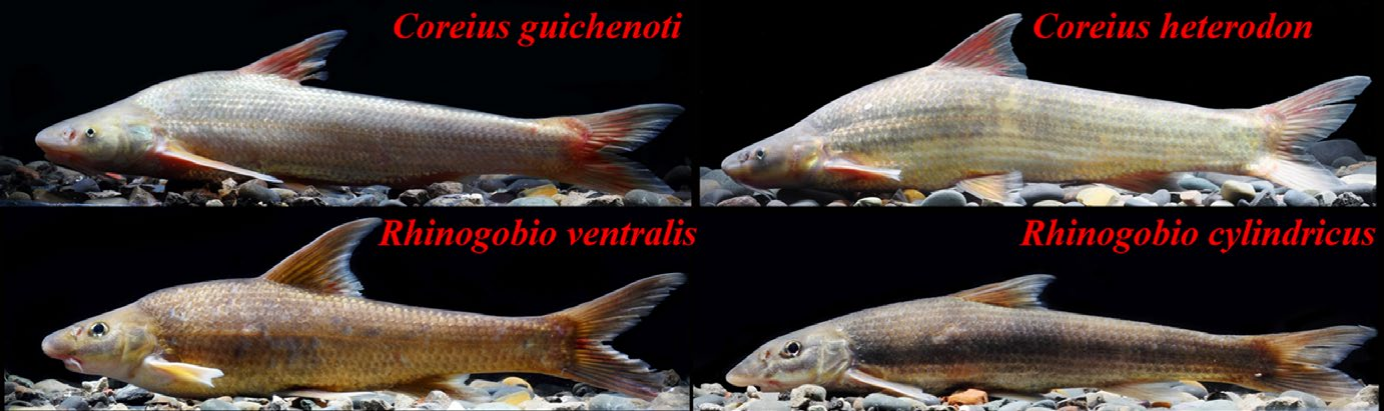
Photos of the four studied gudgeon species

The upper Yangtze River supports the highest biodiversity of the Palearctic region, with 286 fish species distributes in its mainstream and tributaries, and 124 of these species are endemic to this area (He, Wang, Lek, Cao, & Lek‐Ang, 2011; Matthews, 1998; Nelson, 1994). However, little is known about the potential mechanism facilitating the coexistence of these sympatric species. In this article, we studied food resource partitioning among four abundant Gudgeons (Teleostei: Cyprinidae: Gobioninae) in the upper Yangtze River: *Coreius guichenoti*, *Coreius heterodon*, *Rhinogobio ventralis,* and *Rhinogobio cylindricus* (Figure 1). Among them, *C. guichenoti*, *R. ventralis,* and *R. cylindricus* are endemic to the upper Yangtze River. All these species show similar morphological (e.g., elongated body, inferior mouth, and small eyes) and ecological characters (e.g., inhabit running waters, bottom‐feeding, and release pelagic eggs into stream currents; Zeng & Liu, 2011), which provide a unique opportunity to examine the mechanism facilitating the coexistence of sympatric species with similar niches (Wang, Liu, Lin, Yang, & Liu, 2015). Therefore, the present study aims to (a) examine the possible seasonal changes in diet composition and feeding strategy among these sympatric species and (b) better understand the coexistence phenomenon of sympatric species.

## 2 MATERIAL AND METHODS

### 2.1 Study area

**Figure 2:**
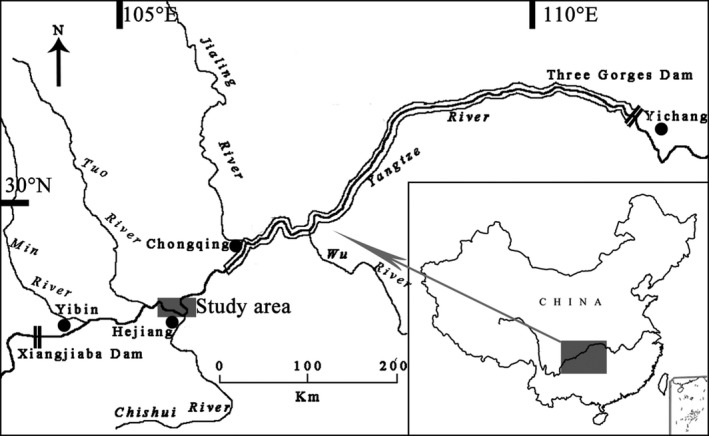
Map of the study area

The Yangtze River is the largest river in China and the third longest river in the world, with a length of ~6,380 km and a drainage area of 1.8 × 10^6^ km^2^ (Hydrology Bureau of Changjiang Water Resources Committee, 2003). The present study was conducted along a 30 km stretch nearby the Hejiang County, Sichuan Province, which is ~100 km upstream of the backwater of the Three Gorges Reservoir (Figure 2). The width of the sampled stretch ranges from 500 to 1,000 m, characterized by a substrate composed of bedrock, boulders, and sand. The maximum water depth measured to ~60 m. The climate is a typical subtropical monsoon, with the air temperature and rainfall vary drastically among seasons. The water temperature usually peaks at ~25.0°C in July or August and drops to ~10.0°C in December or January. The flooding period usually between June and October with the averaged discharge exceed 10,000 m^3^/s, while the dry season occurs from December to May with an averaged discharge of ~3,000 m^3^/s. Liu (2009) surveyed the macroinvertebrate using a bottom D‐net method in the mainstream of the upper Yangtze River. Results showed that macroinvertebrate community in this stretch was dominated by Perlidae, Hydropsychidae, Gammaridae, and Ephemeroptera. Additionally, the diversity and abundance of macroinvertebrate varied significantly with seasons (Liu et al., 2009). In spring, the macroinvertebrate community showed a high diversity and the average density peaked with 165.50 ind/100 m^2^. However, due to the washout of monsoon floods and emergence of some aquatic invertebrates, the diversity of macroinvertebrate reduced significantly in autumn with some groups (e.g., Trichoptera, Diptera, and Neuroptera) almost disappeared from the community, and the average density decreased to only 27.9 ind/100 m^2^. Fish assemblage in this stretch was dominated mainly by the four studied gudgeon species, with the relative biomass reached 60–80% of the total catches collectively (see Liu, Wang, & Cao, 2012 for more details).

## 3 FISH SAMPLING AND DIET EXAMINATION

Sampling for dietary analysis was conducted in spring (April–May) and autumn (August–October) in 2010, generally representing the dry season (low water level, abundant food resource) and the wet season (high water level, low food resource) in this area. Fishes were collected using drift gill net, an active fishing gear, with a mesh size of 3–5 cm (100–200 m long × 1.0–2.3 m high). All samplings were conducted in the daytime. Nine sampling sites were distributed along the study reach with a length of 1–2 km, representing all accessible habitat units (Liu et al., 2012). Specimens were measured to determine standard length (to the nearest mm) and body weight (to the nearest g), and dissected immediately in order to reduce postmortem digestion. The foregut (the section of the intestine from the esophagus to the posterior end of the first loop) was removed and the contents weighted (to the nearest 0.0001 g) and then preserved in 4% formalin for taxonomic assessment and quantification (Herder & Freyhof, 2006). In the laboratory, prey from each gut were identified to the lowest possible taxonomic level under a dissecting microscope, and weighted (to the nearest 0.0001 g) and counted. Unidentifiable prey categories were quantified but not used in further analyses. The cestodes were not quantified because they might be parasites.

### 3.1 Data analysis

Diet composition and feeding strategy of the four species were compared by analyzing the intestinal tract contents. To avoid the possible ontogenetic dietary shifts (Sánchez‐Hernández, Nunn, Adams, & Amundsen, 2018), age‐0 individuals were dismissed and diet analysis included individuals bigger than 96 mm for *C. guichenoti* (Zhou, Zhu, & Liu, 2010), 139 mm for *C. heterodon* (Xu, Deng, Yu, & Wei, 1981), 79 mm for *R. ventralis* (Zhou & He, 1992), and 114 mm for *R. cylindricus* (Ma & He, 2004). Consequently, a total of 936 individuals (size range: 97–334 mm) of the four species were analyzed (Table 1).

**Table 1:** Number and size range of fish specimens used for diet examination (mean ± *SE*; range in parentheses)

Species	Spring		Autumn	
	*n*	Standard length (mm)	*n*	Standard length (mm)
*Coreius guichenoti*	260	175.3 ± 41.0 (106–297)	226	195.4 ± 29.3 (124–334)
*C. heterodon*	65	207.3 ± 22.3 (168–264)	96	238.5 ± 27.0 (175–320)
*Rhinogobio ventralis*	105	156.2 ± 31.7 (97–225)	50	174.2 ± 12.6 (136–201)
*R. cylindricus*	61	185.0 ± 21.7 (143–252)	73	191.8 ± 22.6 (154–292)

The importance of each food category was calculated in terms of frequency of occurrence of prey *F_i_*, relative abundance of prey *A_i_*, and prey‐specific abundance *P_i_* (Amundsen, Gabler, & Staldvik, 1996):

$$F_{i}=N_{i}/N*100$$

$$A_{i}=\sum S_{i}/\sum S_{t}*100$$

$$P_{i}=\sum S_{i}/\sum St_{i}*100$$

where *N_i_* is the number of fishes with prey *i* in their gut, *N* is the total number of fishes with gut contents of any kind, *S_i_* is the total weight of prey *i*, *S_t_* the total foregut content of all foreguts examined, and *St_i_* is the total weight of foregut content with prey *i* in their foregut.

The degree of dietary overlap among each pair of species was calculated using Schoener's (1970) similarity index:

$$D=100-0.5*\sum \left|p_{i}-q_{i}\right|$$

where *p_i_* and *q_i_* represent the proportions by weight of different gut content categories of any two species, respectively. *D* varies between 0 and 1, representing no to complete food overlap. Diet overlap is usually considered significant when *D* exceeds 60% (Wallace, 1981).

To determine diet specialization of each species, diet breadth (*B*) was calculated using Levin's (1968) index:

$$B=1/\sum p_{i}^{2}$$

where *p_i_* is the proportion of each prey *i* in the diet.

The modified Costello (1990) graphical method (Amundsen et al., 1996) was used to assess the seasonal variations in feeding strategy of different fish species. In this method, the prey‐specific abundance (*P_i_*; *y*‐axis) of each prey was plotted against the frequency of occurrence (*F_i_*; *x*‐axis) in a two‐dimensional graph. Information on prey importance, feeding strategy, and phenotype contribution to the niche width can be obtained according to the distribution of points along the diagonals and axes of the diagram. The diagonal from the lower left to the upper right measures the prey importance, with the dominant prey at the top right corner of the diagram and the rare or unimportant prey at the lower left end. The vertical axis indicates the feeding strategy of predator. Fish species that have prey in the upper part of the graph presents a specialized feeding strategy, while species that have all prey in the lower part presents a generalized feeding strategy. The diagonal from the upper left to the lower right represents the phenotype contribution to the niche width. Prey in the upper left part of the graph represents a high BPC (between‐phenotype component), whereas prey in the opposite part represents a high WPC (within‐phenotype component). More interpretation about this method can be obtained from Amundsen et al. (1996).

## 4 RESULTS

### 4.1 Diet composition

A total of 16 prey categories were identified (Table 2). *Limnoperna lacustris* was the most abundant prey for the four studied gudgeons and constituted a large proportion of the diet in both spring and autumn. However, the food composition differed among these species. Crabs were consumed exclusively by *C. guichenoti* constituting 10.04% and 17.14% of the diet in spring and autumn, respectively. Detritus was exploited by *C. guichenoti*, *C. heterodon,* and *R. cylindricus* as the complementary resources in both or a single season, suggesting omnivorous feeding habits of these species. Comparatively, *R. ventralis* fed only on animal prey. Seasonal variations in food composition were also observed. In spring, some species consumed *Macrobrachium*, *Bellamya*, Ephemeroptera, or Chironomidae frequently. However, these prey almost disappeared from their diets in autumn.

**Table 2:** Diet composition of the four studied gudgeon species in both seasons

Prey item	*Coreius guichenoti*				*C. heterodon*				*Rhinogobio ventralis*				*R. cylindricus*			
	Spring		Autumn		Spring		Autumn		Spring		Autumn		Spring		Autumn	
	*F_i_*	*A_i_*	*F_i_*	*A_i_*	*F_i_*	*A_i_*	*F_i_*	*A_i_*	*F_i_*	*A_i_*	*F_i_*	*A_i_*	*F_i_*	*A_i_*	*F_i_*	*A_i_*
*Limnoperna lacustris*	82.86	77.44	65.31	72.41	85.71	80.89	86.54	98.38	91.86	97.97	100	99.37	89.66	89.68	31.25	56.18
Hydropsychidae	3.81	0.76	–	–	6.12	2.84	–	–	–	–	–	–	24.14	9.52	95.31	4.12
Perlidae	12.86	2.42	2.55	0.31	4.08	8.56	–	–	4.65	1.72	–	–	–	–	–	–
*Anax*	1.43	0.08	0.51	0.05	–	–	–	–	–	–	–	–	–	–	–	–
Tubificidae	3.33	0.02	1.02	0.01	6.12	0.02	–	–	4.65	0.01	–	–	–	–	31.25	0.12
*Macrobrachium*	1.9	0.52	–	–	–	–	–	–	–	–	–	–	–	–	–	–
*Gammarus*	–	–	–	–	–	–	–	–	–	–	–	–	3.45	0.02	–	–
Sinopotamidae	7.14	10.04	17.35	17.14	–	–	–	–	–	–	–	–	–	–	–	–
*Bellamya*	3.33	2.04	–	–	8.16	0.91	–	–	–	–	–	–	–	–	–	–
Ephemeroptera	–	–	–	–	–	–	–	–	–	–	–	–	1.72	0.04	–	–
Gerridae	–	–	–	–	–	–	–	–	–	–	–	–	1.72	0.01	–	–
*Sphaerium*	–	–	–	–	2.04	0.02	–	–	5.81	0.29	2.94	0.64	–	–	–	–
Chironomidae	–	–	–	–	–	–	–	–	1.16	0.01	–	–	–	–	–	–
Fishes	–	–	0.51	0.47	–	–	–	–	–	–	–	–	–	–	–	–
Detritus	17.14	6.68	12.76	9.61	10.2	6.75	3.85	1.62	1.16	0.01	–	–	8.62	0.73	42.19	39.59

Notes

*F_i_*, the frequency of occurrence of each prey item; *A_i_*, the relative abundance of each prey item.

### 4.2 Dietary niche breadth

**Figure 3:**
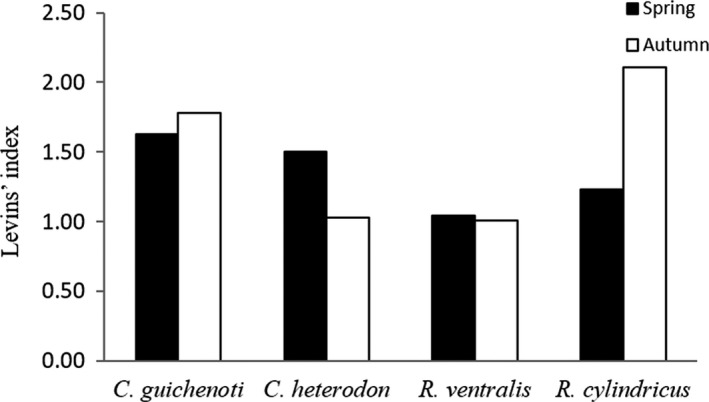
Seasonal changes in dietary niche breadth (Levins’ index) of the four studied gudgeon species

The dietary niche breadth of the studied species varied across seasons. *Coreius guichenoti* and *R. cylindricus* expanded their dietary niche breadth in autumn, while *C. heterodon* and *R. ventralis* reduced their dietary niche breadth (Figure 3).

### 4.3 Diet overlap

Considerable dietary overlap among the four studied gudgeons was detected in spring, with all Schoener's indexes exceeded 60% (Table 3). However, changes were observed in autumn. *Coreius guichenoti* and *R. cylindricus* showed declined dietary overlaps with other species. In contrast, *C. heterodon* and *R. ventralis* showed an extreme high dietary overlap (98.38%) in autumn.

**Table 3:** Diet overlaps (Schoener's index) among the four studied gudgeon species

Species pairs	Spring	Autumn
*C. guichenoti* and *C. heterodon*	87.08	74.13
*C. guichenoti* and *R. ventralis*	78.72	72.51
*C. guichenoti* and *R. cylindricus*	78.93	65.61
*C. heterodon* and *R. ventralis*	82.65	98.38
*C. heterodon* and* R. cylindricus*	84.48	57.80
*R. ventralis* and* R. cylindricus*	89.68	56.18

### 4.4 Feeding strategy

**Figure 4:**
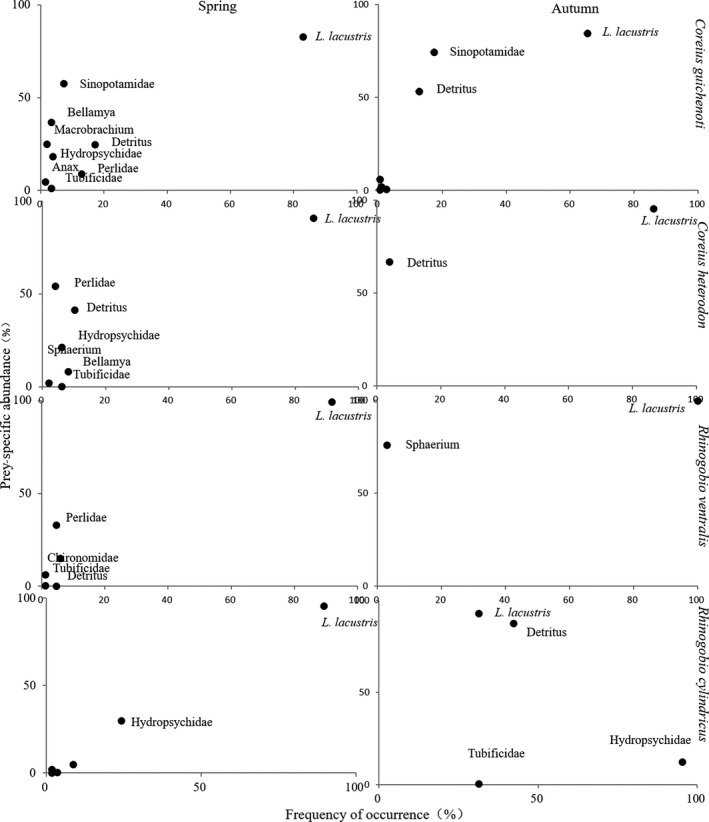
Feeding strategy variations of the four studied gudgeon species

In spring, all studied species exhibited a remarkable similarity in their feeding strategies (Figure 4). Most individuals of these species consumed *L. lacustris* as the most important food resource, while other prey, such as Hydropsychidae, Perlidae, *Anax*, Tubificidae, *Macrobrachium*, *Gammarus*, Sinopotamidae, *Bellamya*, Ephemeroptera, Gerridae, *Sphaerium*, Chironomidae, and detritus, were consumed by less than 20% of examined individuals. However, different types of dietary shift were observed in autumn. Specifically, *C. guichenoti* and *R. cylindricus* reduced the consumption of *L. lacustris* and many individuals exploited other prey, such as detritus, Sinopotamidae, or Hydropsychidae, as important food resources. On the other hand, *C. heterodon* and *R. ventralis* more specialized on *L. lacustris* and seldom ate other prey.

## 5 DISCUSSION

### 5.1 Diet composition

The analysis of dietary composition revealed that the four studied gudgeon species fed predominantly on aquatic macroinvertebrates, such as *L. lacustris*, Hydropsychidae, Perlidae, Tubificidae, Sinopotamidae, *Bellamya*, *Gammarus*, Ephemeroptera, and *Sphaerium*. These results were broadly in line with previous studies (Huang & Deng, 1990; Xu et al., 1981; Zhou & He, 1992). For example, Huang and Deng (1990) found that *C. heterodon* fed mainly on aquatic insects, Chironomidae larvae, and *L. lacustris*. Xu et al. (1981) observed that the diet of *C. Heterodon* was mainly composted of *L. lacustris*, *Sphaerium,* and *Gammarus*. Zhou and He (1992) noted that *L. lacustris*, *Sphaerium,* and aquatic insects were the most important prey categories for *R. ventralis*. Besides, the present study showed that detritus was consumed by *C. guichenoti*, *C. heterodon,* and *R. cylindricus* as an important supplemental food resource, despite of the low nutritional and energetic value (Bowen, 1979, 1987). This phenomenon suggested omnivorous feeding habits of these species. On the other hand, *R. ventralis* fed exclusively on aquatic macroinvertebrates, which might indicate that the species can be considered as obligate carnivore.

*Limnoperna lacustris* was the most important prey for all studied species. Previous studies have demonstrated that the macroinvertebrate community of the upper Yangtze River was mainly composed by Perlidae, Hydropsychidae, Gammaridae, and Ephemeroptera, whereas mollusks constituted a relatively small proportion (Liu, 2009; Liu et al., 2009). Because of the high flow velocity in the upper Yangtze River, it would be very difficult for these benthic‐feeding fish to catch the drift aquatic insects. Therefore, they would prefer to select the benthic, sessile, and relative slow‐moving prey categories, in order to maximize their net rate of energy gain, as predicted by the optimal foraging theory (Emlen, 1966; Gerking, 1994).

Among the four studied species, *C. guichenoti* showed the broadest dietary niche. Some large‐sized prey categories, such as Sinopotamidae, *Macrobrachium,* and fish, were exploited exclusively by *C. guichenoti*. Morphologically, *C. guichenoti* has a relative larger mouth than other species. This large mouth might allow *C. guichenoti* to capture large‐sized prey more efficiently. Thus, the low utilization of crabs, shrimps, fish, and other large‐sized prey by *C. heterodon*, *R. ventralis,* and *R. cylindricus* was probably a result of morphological constraints of feeding apparatus, similar as in other species (Magalhães, 1993).

Seasonal variations in the diet composition of studied fish species were observed. Some aquatic invertebrates, such as *Macrobrachium*, *Bellamya*, Ephemeroptera, and Chironomidae, were commonly consumed by fish species in spring but little consumed in autumn. Many investigations have revealed that the availability of macroinvertebrate in the upper and middle Yangtze River changed significantly across seasons (Chen, Xia, Pan, Xu, & Ni, 2017; Jiang, Xiong, & Xie, 2017; Liu et al., 2009). In spring, the macroinvertebrate showed a high diversity and abundance, benefited from the moderate water temperature and stable flow regime (Jiang et al., 2017; Liu et al., 2009). However, with the washout of monsoon floods and the emergence of some species (e.g., Trichoptera, Diptera, and Neuroptera) in late summer and autumn, the diversity and abundance of aquatic invertebrates both decreased significantly (Jiang et al., 2017; Liu et al., 2009). Therefore, the seasonal changes in diet composition were probably resulted from the decreased availability in the environment, as observed by other studies (Magalhães, 1993; Martin & Genner, 2009).

### 5.2 Trophic partitioning

We observed a considerable high dietary overlap among the studied species, especially in spring. However, high degree of dietary overlap may not always indicate competition (Deus & Petrere‐Junior, 2003; Gabler & Amundsen, 2010; Sánchez‐Hernández et al., 2011). When food resource availability is high, sympatric fish species may become more generalist, which can also result in high dietary overlap (Gabler & Amundsen, 2010). In the present study, the four studied gudgeon species have similar feeding apparatus and all prefer to utilize the same aquatic invertebrate (Zeng & Liu, 2011). Moreover, the macroinvertebrate community commonly shows the highest diversity and density in spring (Liu et al., 2009), which may not lead to interspecific competition for food resource in this season. Therefore, it is possible that the high prey availability in spring enables fish species to share the same food resources, and hence the observed high diet overlap (Gabler & Amundsen, 2010).

However, changes in feeding strategies were observed in autumn. *Coreius guichenoti* and *R. cylindricus* increased their dietary niche breadth and consumed detritus, Sinopotamidae or Hydropsychidae as important complementary food resource, which resulted in declined diet overlap with other species. On the other hand, *C. heterodon* and *R. ventralis* showed an extremely dietary overlap (98.38%) because they both reduced their dietary niche breadth and exploited *L. lacustris* as the exclusive predominated food resource. Thus, we posit that these species undergo strong competition for food during the autumn (Hammerschlag, Ovando, & Serafy, 2010; Jardas, Santic, & Pallaoro, 2004; Tyler, 1971; Gray et al., 1997). Numerous studies have demonstrated that seasonal fluctuation in food resource availability may affect the trophic relationships among sympatric fish species (Sánchez‐Hernández et al., 2016, 2011; Gray et al., 1997). When food resource availability is high, fish species may become more generalist (Gabler & Amundsen, 2010). However, with the decline of food resources, species may specialize or generalize in resource use, according to the extent of food resource limitation (Genner et al., 1999; Amarasekare., 2003; Gabler & Amundsen, 2010; Sánchez‐Hernández et al., 2017). The present study confirmed that interspecific trophic relationships of fish assemblage may be more complex than we have expected. Even in the same community, divergence and convergence in resource use among species can occur simultaneously, as proposed by Genner et al. (1999). In this study, *C. guichenoti* and *R. cylindricus* showed some niche differentiation with other species in autumn when the food availability decreased. However, no significant dietary partitioning between *C. heterodon* and *R. ventralis* was found as they both decreased the dietary niche breadth and exploited *L. lacustris* as the predominated food resource. The coexistence of *C. heterodon* and *R. ventralis* with high overlap may be facilitated by the dietary segregation of *C. guichenoti* and *R. cylindricus*. As the latter two species increased their dietary niche breadth and consumed other prey categories, such as detritus, Sinopotamidae, or Hydropsychidae as important complementary food components, the remained *L. lacustris* may become abundant enough to support the former two species (Deus & Petrere‐Junior, 2003). Furthermore, segregations in microhabitat use (Magalhães, 1993), prey size (Sánchez‐Hernández & Cobo, 2011; Sánchez‐Hernández et al., 2011), diel feeding rhythms (Sánchez‐Hernández et al., 2011), and feeding patches (Hesthagen et al., 2004; Olson et al., 2016; Sánchez‐Hernández et al., 2016) may also alleviate the interspecific competition among sympatric fish species and thereby facilitate their coexistence. Therefore, future studies should pay more attention to these aspects, in order to enhance our understandings of coexistence mechanism of these sympatric gudgeon species in the upper Yangtze River.

## 6 CONCLUSIONS

The present study revealed seasonal differences in food resource utilization among four sympatric gudgeons. High diet overlap among studied species was observed due to their common utilization on the abundant aquatic invertebrate, especially in spring. However, changes in feeding strategies were observed in autumn. Specifically, *C. guichenoti* and *R. cylindricus* increased their dietary niche breadth and presented declined dietary overlaps between other species, while *C. heterodon* and *R. ventralis* reduced their dietary niche breadth and specialized on *L. lacustris*. These results corroborated that the food resource partitioning among sympatric fish species may fluctuate with seasons, in order to reduce the possible interspecific competition for food resources. Noteworthy, this study relied on previous studies about the macroinvertebrate community of the study area (Liu, 2009; Liu et al., 2009) to discuss the seasonal changes in food resource partitioning. Additionally, the present study was focused only on the four most dominated species. In order to have a comprehensive understanding of the interspecific relationships of the whole fish assemblage, more attentions should be put into the changes of food availability and more species should be included in further studies. Despite the above‐mentioned problems, the present study provides valuable information for understanding the resource partitioning of sympatric species in the upper Yangtze River.

## CONFLICT OF INTEREST

None declared.

## AUTHOR CONTRIBUTIONS

Fei Liu contributed to field sampling, data analyses, and writing of the manuscript. Jianwei Wang and Huanzhang Liu contributed to research design and writing of the manuscript.

## Data Availability

The data supporting the results, such as the number and size range of fish specimens and the diet composition of the studied fish species, have been listed in the tables.
